# Identification of a panel of sensitive and specific DNA methylation markers for squamous cell lung cancer

**DOI:** 10.1186/1476-4598-7-62

**Published:** 2008-07-10

**Authors:** Paul P Anglim, Janice S Galler, Michael N Koss, Jeffrey A Hagen, Sally Turla, Mihaela Campan, Daniel J Weisenberger, Peter W Laird, Kimberly D Siegmund, Ite A Laird-Offringa

**Affiliations:** 1 Departments of Surgery and of Biochemistry and Molecular Biology, Norris Cancer Center, Keck School of Medicine, University of Southern California, Los Angeles, CA, 90089-9176, USA; 2 Department of Pathology, Keck School of Medicine, University of Southern California, Los Angeles, CA, 90089-9092, USA; 3 Department of Surgery, Keck School of Medicine, University of Southern California, Los Angeles, CA, 90089-9202, USA; 4 Department of Preventive Medicine, Keck School of Medicine, University of Southern California, Los Angeles, CA, 90089-9175, USA

## Abstract

**Background:**

Lung cancer is the leading cause of cancer death in men and women in the United States and Western Europe. Over 160,000 Americans die of this disease every year. The five-year survival rate is 15% – significantly lower than that of other major cancers. Early detection is a key factor in increasing lung cancer patient survival. DNA hypermethylation is recognized as an important mechanism for tumor suppressor gene inactivation in cancer and could yield powerful biomarkers for early detection of lung cancer. Here we focused on developing DNA methylation markers for squamous cell carcinoma of the lung. Using the sensitive, high-throughput DNA methylation analysis technique MethyLight, we examined the methylation profile of 42 loci in a collection of 45 squamous cell lung cancer samples and adjacent non-tumor lung tissues from the same patients.

**Results:**

We identified 22 loci showing significantly higher DNA methylation levels in tumor tissue than adjacent non-tumor lung. Of these, eight showed highly significant hypermethylation in tumor tissue (p < 0.0001): GDNF, MTHFR, OPCML, TNFRSF25, TCF21, PAX8, PTPRN2 and PITX2. Used in combination on our specimen collection, this eight-locus panel showed 95.6% sensitivity and specificity.

**Conclusion:**

We have identified 22 DNA methylation markers for squamous cell lung cancer, several of which have not previously been reported to be methylated in any type of human cancer. The top eight markers show great promise as a sensitive and specific DNA methylation marker panel for squamous cell lung cancer.

## Background

Cancer is responsible for one in four deaths in the US, making it the second most common cause of death [1]. Lung cancer is the leading cancer killer in men and women.

Over 160,000 Americans will die of this disease in 2007. In men, lung cancer accounts for 31% of cancer deaths, killing more men than leukemia and prostate, colorectal, and pancreatic cancer combined. In women, lung cancer accounts for 27% of all cancer deaths, taking as many lives as breast and colorectal cancer combined [1]. The overall five-year survival rate of lung cancer patients is 15%, significantly lower than that of patients with prostate cancer (99.9%), breast cancer (88.5%) or colon cancer (64.1%) [1]. This rate increases dramatically to greater than 50% when lung cancer is diagnosed at an early stage. However, only 14–16% of cases are detected early [1].

In contrast to breast, colon, and prostate cancer, no routine screening method for early detection of lung cancer exists. Methods based on imaging (chest X-ray, low dose spiral computed tomography (LDSCT), autofluorescence bronchoscopy (AFB)), and sputum cytology have been tested, however, none have proven ideal. Screening *via *chest X-ray is not sufficiently sensitive [2], and trials demonstrated that its use in high risk populations showed no decrease in mortality [3]. LDSCT screening can detect a number of stage I lung cancers, with survival at 10 years reported as high as 88% [4]. However, the possibility of lead-time bias and the high false positive rate [5] limit the utility of this screening modality. These false positive tests frequently lead to invasive procedures to remove lesions that later prove to be benign [6]. In addition, LDSCT appears to favor detection of peripheral lesions, being less effective at detecting small pre-invasive/micro-invasive lesions in the central airways [7]. Its effects on reducing lung cancer mortality remain in question [8]. Autofluorescence bronchoscopy (AFB) also has a high false positive rate [9,10], and preferentially detects centrally located cancers. Screening by sputum cytology can detect a number of aspymptomatic cases, but it has not been shown to decrease lung cancer mortality [11]. Studies using molecular marker techniques on sputum samples appear promising [12].

Given the poor five-year survival rates and limitations of current screening techniques, it is clear that improved methods for early detection of lung cancer are needed. One strategy is to develop sensitive and specific molecular markers that distinguish cancer type and subtype, that are detectable in 'remote' patient media (e.g. blood, sputum) by non-invasive/minimally invasive means, and that can be assayed using a quantitative approach.

DNA methylation has emerged as a prime source of potential cancer-specific biomarkers. In cancer, despite global DNA hypomethylation, many genes become hypermethylated. Typically this occurs in CpG rich regions called CpG islands at/near gene promoters. Methylation often results in the silencing of tumor suppressor or growth regulatory genes [13]. Such cancer-specific hypermethylation results in differential DNA methylation profiles between tumor and non-tumor tissues, which can be exploited to distinguish the two, allowing DNA methylation to serve as a cancer-specific molecular marker. Using bisulfite treatment, which embeds methylation information in the DNA sequence, coupled with a sensitive and quantitative real-time PCR-based assay (MethyLight), hypermethylated CpGs form stable, easily amplifiable, and readily available biomarkers [14]. As no one locus can be expected to detect all cancers of a particular type, reactions for multiple loci can be easily combined into panels of markers, increasing the potential to detect lung cancer in a highly sensitive and specific manner. Because our end goal is a non-invasive lung cancer detection method using DNA methylation markers, it is worth noting that DNA hypermethylation has been detected in remote patient media such as sputum, blood [15] and bronchoalveolar lavage (BAL) [16] from lung cancer patients.

Lung cancer is divided clinically into two major subtypes – the rapidly progressing small cell lung cancer (SCLC), and the more common non-small cell lung cancer (NSCLC). As NSCLC accounts for > 85% of all lung cancer cases, and is less aggressive than SCLC, there is a greater chance for early detection, resulting in increased patient survival. NSCLC is divided into four major histological subtypes: adenocarcinoma (AD), squamous cell carcinoma (SQ), large cell carcinoma and others (carcinoids, neuroendocrine cancers, etc). A comparison of SQ and AD of the lung shows differences in DNA hypermethylation profiles [17-19], in expression of therapeutic targets [20], in the mutational and polymorphic spectra [21,22] and in gene expression profiles [23]. The region of the lung in which these tumors usually occur also differs, with AD typically located at the periphery and SQ arising near the central airways. Given the distinct nature of SQ and AD, it is to be expected that different molecular markers would need to be developed to sensitively detect these two types of lung cancer. We have recently identified a panel of DNA methylation markers for lung adenocarcinoma [24]. Here we focus on the development of molecular markers for squamous cell lung cancer.

SQ accounts for 25 – 35% of all lung cancer cases in the United States [25]. Our goal was to identify a panel of DNA markers that are frequently and highly methylated in SQ lung tumors when compared to non-tumor lung. Such a panel may be used for non-invasive/minimally invasive and potentially subtype-specific early detection of SQ lung cancer. We envision that in the future, detection of DNA methylation markers in remote media (blood, sputum, bronchoalveolar lavage) might complement less specific imaging-based lung cancer screening tests, and if sensitivity and specificity are high enough, might eventually be directly applied to the screening of high risk populations.

## Results

In an effort to develop sensitive and specific molecular markers for squamous cell carcinoma (SQ) of the lung, the methylation status of 42 candidate loci was examined in a collection of 45 tumors and histologically normal adjacent non-tumor lung samples from the same patients. These 42 loci were identified in a pre-screen examination of the methylation status of 304 MethyLight reactions on cell lines and a small number of tumors distinct from the ones used in this study (data not shown). As our aim was to identify novel high penetrance markers for lung SQ, many loci previously reported as methylated in NSCLC/SQ were not included in our study due to their lower methylation frequency. In five of the 42 loci (*HRAS, MGMT, MTHFR, PAX8 *and *SLC38A4*), the region examined is not in a CpG island. In our pre-screen, multiple reactions in and around the CpG islands of these loci were tested and the chosen reactions showed the highest methylation in cancer. Paired histologically normal adjacent lung tissue samples, derived from a separate non-cancer block of the lung cancer patients, were used as control samples. Thus, our control tissue matched tumor tissue fully with respect to most variables, including environmental exposures, age, gender, ethnicity and genetic background. The use of paired control tissue from lung cancer patients, which may show higher background methylation, ensures the identification of markers that are hypermethylated in a *cancer-specific *manner. MethyLight provides a quantitative measure for methylation at each locus; the percentage of methylated reference (PMR) value reflects the level of DNA methylation at the locus examined compared to *in vitro *methylated control DNA.

**Figure 1:**
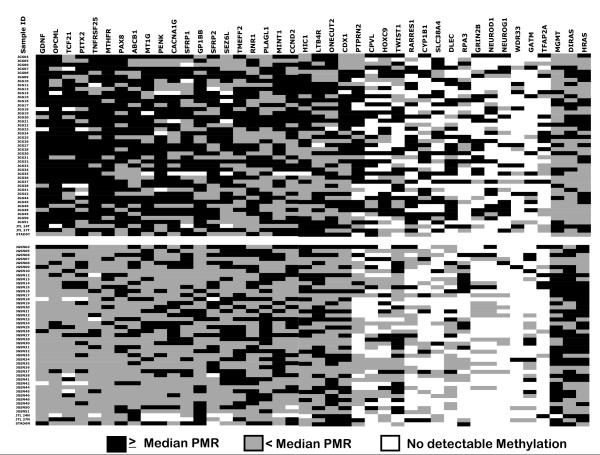
Schematic representation of DNA methylation levels of 42 loci in 45 tumor and adjacent non-tumor squamous cell lung cancer cases. Black indicates high methylation levels (≥ median PMR of all, tumor and non-tumor, positive samples). Grey indicates low methylation (< median of the positive PMR values), and white indicates no detectable methylation.

We observed a high methylation frequency (the fraction of samples showing any methylation) for all 42 loci in both the tumors and the adjacent non-tumor tissues taken from the same patient (Figure 1, Table 1). The DNA methylation in histologically normal adjacent non-tumor lung is likely due, on the one hand, to the sensitivity of MethyLight, and on the other, to age and/or environmental exposure, and has been observed in other studies [26-28]. We examined the statistical significance of differences in DNA methylation levels in tumor *versus *adjacent non-tumor tissue using the PMR as a continuous variable. Out of the 42 loci studied, 13 were previously reported to be methylated in NSCLC. Hence, a marker from these 13 was considered statistically significant if it attained the 0.05 level of significance without correction for multiple testing. A marker from the remaining 29 targets was declared statistically significant if it exceeded the 5% false-discovery rate threshold defined using the Benjamini and Hochberg [29] approach. Overall, twenty-five of the 42 loci examined showed a statistically significant difference (highlighted in italics in Table 1). Three markers – *DIRAS3*, *MGMT*, and *HRAS *– showed statistically significant hypermethylation in *non-tumor *tissue. The importance of this suggested loss of methylation in the tumors was not further explored here, as we are focused on identifying positive methylation markers for SQ of the lung. The phenomenon could be of interest for future studies. The remaining 22 loci were found to be statistically significantly hypermethylated in the tumors (Table 1). This is the first report of methylation in any cancer for five loci (*CPVL, HOXC9, PAX8, PTPRN2*, and *SLC38A4*), flagging these loci as potential novel cancer markers. Eight loci (*GDNF, MTHFR, OPCML, TNFRSF25, TCF21, PAX8, PTPRN2*, and *PITX2*) showed highly statistically significant differences with p-values <0.0001.

**Table 1:** Statistical analysis of differences in methylation levels between tumor and adjacent non-tumor tissues

	**Tumor**		**Adjacent Non-Tumor**			
**Gene Name**^a^	**Methylation** **Frequency**^b^	**Median** **PMR**^c^	**Methylation** **Frequency**^b^	**Median** **PMR**^c^	**p-value**^d^	**MC** **Corr.**^e^

***GDNF***	*95%*	*67.11*	*95%*	*3.29*	*5.0E-11*	*0.0017*
***MTHFR***^f^	*100%*	*56.51*	*100%*	*25.95*	*2.0E-10*	*
***OPCML***^g^	*95%*	*19.49*	*98%*	*5.80*	*1.0E-09*	*
***TNFRSF25***	*98%*	*50.52*	*93%*	*25.89*	*2.0E-07*	*
***TCF21***	*93%*	*60.64*	*88%*	*11.17*	*3.0E-07*	*
***PAX8***^f^	*100%*	*83.90*	*100%*	*69.49*	*9.0E-06*	*0.0034*
***PTPRN2***	*80%*	*35.60*	*58%*	*4.02*	*1.0E-05*	*0.0052*
***PITX2***	*93%*	*19.37*	*95%*	*1.50*	*3.0E-05*	*0.0069*
*MT1G*	*95%*	*1.89*	*93%*	*0.59*	*0.0001*	*0.0086*
*PENK*	*93%*	*14.30*	*95%*	*6.82*	*0.0002*	*0.0103*
*GP1BB*	*98%*	*59.52*	*100%*	*42.68*	*0.0009*	*
*MGMT*^f^	*100%*	*26.73*	*98%*	*33.96*	*0.0009*	*
*SLC38A4*^f^	*56%*	*6.00*	*31%*	*0.00*	*0.0010*	*0.0121*
*SFRP2*	*98%*	*10.19*	*91%*	*2.67*	*0.0038*	*
*RARRES1*	*60%*	*9.27*	*47%*	*0.71*	*0.0048*	*0.0138*
*DIRAS3*	*100%*	*57.25*	*100%*	*63.92*	*0.0074*	*
*NEUROG1*	*37%*	*3.03*	*17%*	*0.08*	*0.0079*	*0.0155*
*WDR33*	*31%*	*0.34*	*9%*	*0.20*	*0.0089*	*0.0172*
*TFAP2A*	*46%*	*5.68*	*20%*	*10.00*	*0.0092*	*0.0190*
*SFRP1*	*91%*	*1.63*	*98%*	*0.56*	*0.0124*	*
*CYP1B1*	*58%*	*3.53*	*40%*	*0.41*	*0.0143*	*
*HOXC9*	*66%*	*10.62*	*60%*	*0.48*	*0.0157*	*0.0207*
*ABCB1*	*100%*	*27.07*	*100%*	*24.33*	*0.0193*	*0.0224*
*HRAS*^f^	*100%*	*82.08*	*100%*	*90.01*	*0.0215*	*0.0241*
*GRIN2B*	*44%*	*28.03*	*31%*	*1.26*	*0.0235*	*0.0259*
CACNA1G	93%	0.74	95%	0.44	0.0282	0.0276
HIC1	98%	33.81	100%	23.52	0.0315	0.0293
CPVL	66%	1.65	53%	0.33	0.0739	0.0310
SEZ6L	89%	5.08	95%	3.51	0.0745	0.0328
NEUROD1	44%	9.97	33%	0.70	0.0748	0.0345
CCND2	98%	1.74	96%	1.18	0.0827	0.0362
MINT1	98%	3.18	98%	2.49	0.0879	0.0379
DLEC	55%	19.39	64%	0.17	0.1169	*
RNR1	100%	43.28	100%	31.91	0.1930	0.0397
BLT1	98%	28.01	100%	25.63	0.2420	0.0414
ONECUT2	98%	17.23	100%	14.29	0.3369	*
PLAGL1	100%	45.72	100%	50.38	0.3747	0.0431
GATM	26%	11.95	69%	0.10	0.4462	0.0448
CDX1	98%	46.96	100%	44.40	0.7469	0.0466
TWIST1	66%	1.98	86%	1.07	0.7578	0.0483
TMEFF2	100%	11.54	100%	11.82	0.7591	*
RPA3	49%	0.24	49%	0.16	0.8863	0.0500

^a ^Human Genome Organization nomenclature. Loci showing a statistically significant difference in methylation between tumor and non-tumor tissue are highlighted in italics. The top eight loci are noted in bold. Loci are ranked in order of ascending p-value. ^b ^Percentage of samples with positive methylation value. ^c ^Median percent methylated reference calculated from positive methylation values. ^d ^p-value calculated by Wilcoxon signed rank test. ^e ^To minimize the risk of false discovery, a false-discovery rate threshold using the Benjamini and Hochberg (1995) approach was applied to all loci not previously found to be methylated in squamous cell lung cancer (see methods). ^f ^This reaction is not targeted to a CpG Island ^g ^This primer/probe set shares homology with the CpG island of the adjacent HNT gene, which appears to have arisen via gene duplication. * Denotes loci previously reported to be methylated in lung cancer tumor samples

Potential biomarkers should be effective in all patients regardless of cancer stage, age, gender or ethnicity. We examined DNA methylation levels in tumors vs. adjacent non-tumor tissue in relation to tumor stage. Because the number of cases was not very large, we grouped stage IA and IB cases together (six IA and twenty-five IB), and stages II and III (no IIA, seven IIB and five IIIA). Each of the eight highly significant loci showed higher DNA methylation levels in tumors vs. adjacent non-tumor lung in both early (stage I; n = 31, p-value range = 1 × 10^-7 ^- 0.0041) and advanced (stage II/III; n = 12, p-value range = 6 × 10^-5 ^- 0.0194) lung cancer patients. When analyzing each stage (IA, IB, IIB and IIIA) independently, the two most significant markers (GDNF and MTHFR) showed significantly higher DNA methylation levels in tumor vs. adjacent non-tumor in every stage, despite the modest number of cases. Comparison of DNA methylation levels for the top eight markers in early vs. advanced cancers showed no significant differences between the methylation levels in these tumors, reinforcing the idea that these markers are not stage-specific. This is important, since effective DNA methylation markers for SQ lung cancer must function on every stage of cancer, but particularly on early stage tumors.

We also examined methylation in tumors in relation to age. *HOXC9 *showed higher levels of DNA methylation in patients under the median age (70: p = 0.021) and *TCF21 *showed increased DNA methylation in females (p = 0.047). However, if a multiple comparisons correction were applied, these differences would not be significant. DNA methylation of *PAX8 *appeared higher in males (p = 0.001; significant even with application of a multiple comparison threshold), a factor that might require consideration if it were to be developed for clinical use. As our population is primarily Caucasian, we were not able to examine DNA methylation levels in relation to ethnicity. Studies are in progress in a larger more ethnically diverse population, to examine the possible relationship of DNA methylation to ethnicity.

**Figure 2:**
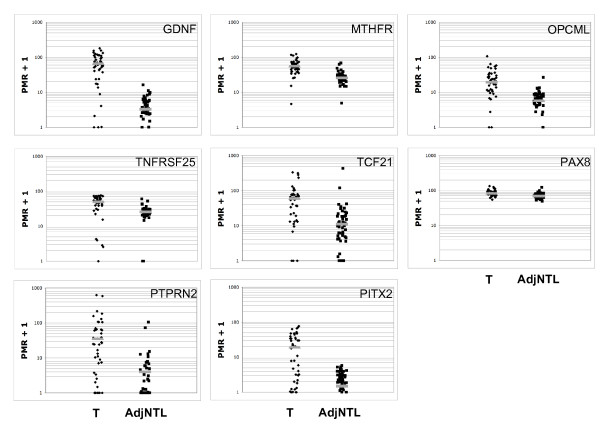
Distribution of PMR values for the eight most significant loci (as ranked by p-value). In each panel, PMR values (indicated by black dots) for tumor (T) are on the left and adjacent non-tumor lung (AdjNTL) on the right. Grey bars represent the median PMR values for tumor and normal tissues.

To provide more insight into the distribution of DNA methylation levels in the tumor and non-tumor samples, we plotted the distribution of PMR values for tumor and non-tumor tissues for the eight most highly significant loci (Figure 2). These plots illustrate differences in the nature of these markers that are not evident from the p-values. For example, *GDNF *appears to promise substantial specificity and sensitivity due to frequently highly elevated DNA methylation of this locus in tumor tissues. A similar pattern is seen in *MTHFR*, *OPCML*, and *TNFRSF25*. For *TCF21*, *PTPRN2*, and *PITX2*, the DNA methylation levels of tumor tissues show a wider distribution and more overlap with non-tumor samples. The *PAX8 *DNA methylation values were tightly clustered, and while the difference is highly statistically significant (p = 9 × 10^-6^), the fold-difference is small, indicating that this marker may not be as useful in the clinical setting.

**Figure 3:**
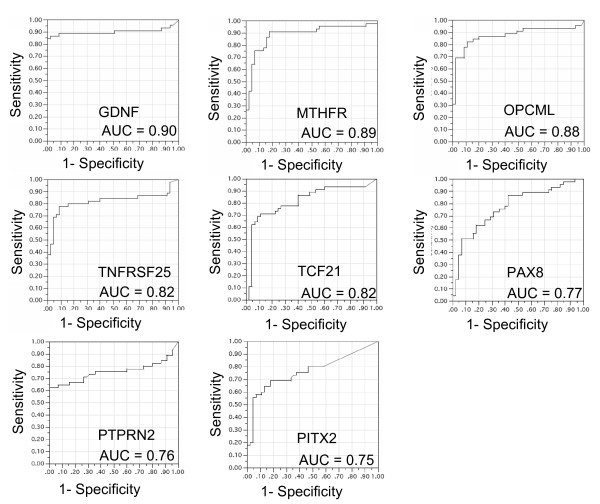
Receiver Operating Characteristic (ROC) curves for the eight most significant DNA methylation markers (as ranked by p-value), using the current collection of tumor and adjacent non-tumor samples.

The utility of clinical markers is often evaluated by generating a receiver operating characteristic (ROC) curve, in which sensitivity *versus *1-specificity at all possible cutoff values is plotted. Ultimately, such ROC curves will be generated based on methylation values detected in remote media. However, here we used ROC curves based on the tumor and non-tumor PMR values to provide an early indication of the potential of the top eight loci as cancer-specific markers. The area under the curve (AUC), an indicator of marker performance, ranged from a modest 0.75 for *PITX2 *to a much better 0.9 for *GDNF *(Figure 3). The sensitivity and specificity values for each of the eight top loci were individually calculated using the present tumor collection in a five-fold cross validation (Table 2). The quantitative marker values were dichotomized at a level that would minimize the classification error. Sensitivity ranged from 58–89% and specificity from 69–100%.

**Table 2:** AUC, Sensitivity & Specificity Analysis

		**5-fold cross validation**	
**Locus**	**AUC**	**Sensitivity**	**Specificity**

GDNF	0.90	0.82	0.98
MTHFR	0.89	0.89	0.82
OPCML	0.87	0.76	0.89
TNFRSF25	0.82	0.76	0.91
TCF21	0.82	0.62	0.91
PAX8	0.77	0.64	0.69
PTPRN2	0.76	0.58	0.89
PITX2	0.75	0.60	1.00

While measurements for several individual markers look promising, it is unrealistic to expect detection of all cases of a particular type of cancer using a single biomarker. Thus, our goal is to develop a *panel *of DNA methylation markers that, used in combination, can sensitively and specifically detect lung SQ. To assess the performance of combinations of our markers in the identification of tumors, we fit a random forest classifier to the data set, using 90 samples and 42 variables. Using bootstrap samples of the data, we grew a forest of 30,000 trees. Splits were determined using a random sample of five variables and trees were grown until there was only one observation in each leaf. When the 42 loci were ranked using the random forests classifier, the top four loci were the same as when the data was ranked by p-value or AUC value, and the order of the ranking is the same for these top four in all three groups (data not shown). Using all 42 loci in combination, we observed 97.7% sensitivity and 97.7% specificity. While this is encouraging, 42 loci are too many to test in a clinical setting. Trimming the panel down to just the top eight loci resulted in 95.6% sensitivity and specificity. Further restricting our analysis to the four most highly ranked loci maintained sensitivity at 95.6% while specificity dropped to 93.3%.

## Discussion

Thirteen of the 42 loci examined here were previously reported to be methylated in lung cancer tumor samples. Consistent with the literature, eight loci (*MTHFR, OPCML, TNFRSF25, TCF21, SFRP2, SFRP1, CYP1B1, GPIBB, DLEC *and *ONECUT2*) [17,24,30-39] are hypermethylated in tumor tissue in our study. Indeed, *MTHFR, OPCML, TNFRSF25 *and *TCF21 *show highly statistically significant differences (p < 1 × 10^-6^) between tumor and adjacent non-tumor tissues in our study. The results for three loci are in contrast with the published literature. *MGMT, DIRAS3 *(previously described as *ARHI*) and *TMEFF2 *(previously described as *HPP1*) have been reported to be hypermethylated in lung cancer [17,18,28,33-36,40-45]. We found that *MGMT *and *DIRAS3 *were statistically significantly more highly methylated in adjacent non-tumor than in SQ samples, while for *TMEFF2*, we observed almost no difference in methylation levels between tumor and non-tumor tissue (Table 1). The differences between our results and the published literature may be due to a variety of reasons, including technical differences (such as the use of the quantitative MethyLight *versus *qualitative methylation specific PCR, or the less sensitive CpG island microarrays), the sampling of a different region of the gene, differences in the lung cancer histologies studied (many studies contain a mix of NSCLC samples), and ethnic/racial differences in the patient populations studied. In the case of MGMT we sampled regions in and out of the CpG island in our pre-screen, and the region outside of the CpG island looked more promising, and was therefore tested. Thus, the primer/probe set we used differs from what has been published in the literature.

When examining the function of the 22 statistically significant potential markers for SQ, four major functional categories emerged. Eight loci encode proteins involved in signaling and growth regulation, seven loci encode transcription factors, four loci encode proteins with metabolic function, and three loci belong to no particular group (Table 3). Our strongest potential biomarkers, the eight most statistically significantly hypermethylated loci, are scattered across the first three of these groups. Because our focus is development of DNA methylation markers, our primary concern is consistent methylation of a particular locus, not whether the associated gene is actually silenced by methylation. Hence, genes in which the consistently hypermethylated locus is outside of the CpG island can serve as markers (e.g. *HRAS, MGMT, MTHFR, PAX8, SLC38A4*), even though the DNA methylation may not be of functional significance. While we have not determined whether the genes for our eight top markers are silenced, there is published evidence for the inactivation of some of these genes in lung cancer. For others, their expression in cancer has not yet been investigated, and might be worth examining in future, more mechanistic, studies. As six of the top eight loci show potentially *functionally *relevant DNA hypermethylation in tumors, we will discuss what is known about their role in cancer development.

**Table 3:** Putative biological role of the 22 statistically significantly hypermethylated loci

**Functional ** **Categories**	**Gene ** **Symbol**^a^	**Gene Name**^b^	**Gene Function**^b^
**Signaling**	GDNF	glial cell derived neurotrophic factor	Growth factor
	GP1BB	glycoprotein I b, beta polypeptide	Platelet membrane receptor
	OPCML	opioid binding protein/cell adhesion molecule-like	Cell adhesion molecule
	PENK	proenkephalin	Opioid peptide precursor
	PTPRN2	protein tyrosine phosphatase, receptor type, N polypeptide 2	Phoshatase
	SFRP1	secreted frizzled-related protein 1	Wnt Signaling modulator
	SFRP2	secreted frizzled-related protein 2	Wnt signalling modulator
	TNFRSF25	tumor necrosis factor receptor superfamily, member 25	Cell surface receptor

**Transcription Factor**	HOXC9	homeobox C9	Transcription factor
	NEUROD1	neurogenic differentiation 1	Transcription factor
	NEUROG1	neurogenin 1	Transcription factor
	PAX8	paired box gene – 8	Transcription factor
	PITX2	paired-like homeodomain transcription factor 2	Transcription factor
	TFAP2A	transcription factor AP 2 alpha	Transcription Factor
	TCF21	transcription factor 21	Transcription factor

**Metabolism**	CYP1B1	cytochrome p450 family 1, subfamily B, polypeptide 1	Liver metabolism
	MT1G	metallothionein 1G	Heavy metal binding
	MTHFR	5,10 methylenetetrahydrofolate reductase (NADPH)	Methyl group metabolism
	SLC38A4	solute carrier family 38, member 4	Amino acid transporter

**Other**	ABCB1	ATP-binding cassette, sub-family B (MDR/TAP), member 1	Drug efflux pump
	RARRES1	retinoic acid receptor responder 1	Unclear
	WDR33	WD repeat domain 33	Unclear

^a ^Gene symbol is the Human Genome Organization nomenclature. ^b ^Gene Name and Function as listed per [63].

*OPCML, TNFRSF25 *and *TCF21 *have been previously reported to be hypermethylated in lung cancer [30-32] and based on their function, methylation-induced silencing could favor tumor growth. Opioid binding protein/cell adhesion molecule (*OPCML*) is an opioid receptor and is involved in cell-cell adhesion. It binds opioid peptides (e.g. enkephalin) and causes apoptosis of lung cancer cell lines, indicating it functions as a tumor suppressor gene. This inhibition was reversed by nicotine [46], which may be of particular interest in lung cancer pathogenesis. It is of note that *PENK*, which encodes the precursor peptide of the *OPCML *ligand enkephalin, was also found to be significantly hypermethylated in tumor tissue in our studies. This might suggest methylation-induced silencing of a tumor suppressor pathway. We recently reported *OPCML *as highly methylated in lung adenocarcinoma, [24] indicating that it is a potential AD/SQ lung cancer biomarker.

Tumor necrosis factor receptor superfamily member 25 (*TNFRSF25*) has been shown to be methylated in bladder cancer, and very recently methylation in lung SQ was reported [31,47]. As this receptor mediates apoptosis, methylation-induced silencing may facilitate evasion of cell death – a key step in cancer growth. The transcription factor *TCF21 *has been reported to be more highly methylated in lung cancer tissue than non-tumor adjacent lung, and overexpression in mouse xenografts results in a reduction in tumor size and weight [32]. This implies a tumor suppressor function for *TCF21*, therefore tumor-associated promoter DNA methylation, and possibly transcriptional silencing, are not surprising.

For other genes, such as *PITX2, PAX8 *and *PTPRN2*, the biological consequences of DNA methylation remain a question. Functionally, it is unclear how *PITX2 *silencing would contribute to lung cancer growth. This member of the paired-like homeodomain transcription factor family functions in left-right asymmetry in development [48], but has no described function in adult lung. However, cancer-related methylation is reported in other tissues in which the gene has no described function, for example, in acute myeloid leukemia [49], breast cancer [50], and prostate cancer [51]. Interestingly, higher DNA methylation levels of *PITX2 *are associated with greater recurrence of both breast and prostate cancer [50,51]. Whether such a link exists in lung cancer will require further studies. Protein tyrosine phosphatase, receptor type, N polypeptide 2 (*PTPRN2*) is an autoantigen involved in insulin dependent diabetes mellitus [52]. No previous reports of methylation of PTPRN2 exist, making it a potentially novel cancer biomarker.

The most intriguing of the identified loci is the top marker *GDNF*, encoding glial cell line-derived neurotrophic factor. *GDNF *has been reported to be overexpressed in lung tumor tissue [53] and is silent in normal adult lung [54]. As a ligand for the RET proto-oncogene, *GDNF *would be a likely candidate for promoting cancer progression, and has been proposed to do so in pancreatic cancer [55]. DNA methylation of this locus would seem contradictory. However, the high DNA methylation we report is at promoter 2 (located at the intron 1/exon 2 boundary of *GDNF*), a promoter that has been shown to have low activity [56]. Indeed, in our preliminary studies, a primer designed against the primary promoter of *GDNF *showed no hypermethylation (data not shown). It may be possible that DNA methylation at the downstream promoter is somehow related to the transcriptional activity from the upstream promoter. Given the fact that *GDNF *is, to our knowledge, the strongest candidate DNA methylation marker for lung SQ identified to date, this issue would be worth investigating further.

While the top eight markers identified in this study show highly significant DNA hypermethylation in cancer, it will of course be important to validate these markers in an independent collection of samples. Such studies are in progress using a specimen collection balanced for gender and the major ethnic groups in the United States.

## Conclusion

Our primary goal is to find sensitive and specific biomarkers for the early detection of lung cancer. Differences in the biology and treatment of different lung cancer histological subtypes warrant the development of markers for each cancer subtype. We have recently reported a panel of DNA methylation markers for lung adenocarcinoma [24]. Here we report the identification of promising DNA methylation markers for squamous cell lung cancer. Statistical analysis of the difference in DNA methylation levels between SQ tumor and adjacent non-tumor lung tissue identified 25 statistically significant loci. Of these, three are potential negative DNA methylation markers (more methylated in adjacent non-tumor tissues), while 22 are potential positive DNA methylation markers. Of the 22 loci, we focused on those eight that were ranked most significantly hypermethylated in the cancer versuspaired non-cancer samples by p-value and ROC curves. These eight loci are significantly hypermethylated in both early (stage I) and more advanced cancers. Two of those eight loci (*PAX8, PTPRN2*) have never been reported to be hypermethylated in human cancer specimens, and thus constitute promising new candidate cancer markers. To our knowledge, the eight-locus panel consisting of *GDNF, MTHFR, OPCML, TNFRSF25, TCF21, PAX8, PTPRN2 *and *PITX2*, constitutes the highest sensitivity and specificity DNA methylation marker panel for lung SQ reported to date. Following its validation on a separate set of tumor and non-tumor lung samples, the next step will be to examine the DNA methylation of these loci in remote media (such as blood, sputum, bronchoalveolar lavage) from lung cancer patients and control non-cancer cases. In conjunction with our work on AD lung cancer and ongoing studies of other NSCLC subtypes, we hope to develop a panel of markers for the sensitive and specific detection of non-small cell lung cancer that would also identify the histological subtype. The further development of DNA methylation markers promises to be important not only for diagnostics, but also for prognostication, the ability to follow response to therapy, and guidance in the choice of treatment.

## Methods

### Tissue samples and DNA extraction

Samples were collected from the Los Angeles County Hospital archives, the Norris Comprehensive Cancer Center archives and the National Disease Research Interchange (NDRI). Study subjects included 21 males and 22 females ranging in age from 45 – 84 at time of diagnosis (median age: 70 years old). Age and gender information was missing for 2 patients. The study population was primarily Caucasian, with 35 Caucasians, 2 African Americans and race unknown for 8 patients. Information as to tumor stage was available for 43 of the 45 patients. TNM status was either listed in the pathology report, or discerned from the report using the International System for Staging Lung Cancer [57]. This information was used to assign tumor stage. There were 6 stage IA, 25 stage IB, 7 stage IIB and 5 stage IIIA patients. Sections were cut from separate, histologically verified, tumor and adjacent non-tumor paraffin blocks. A 5 μm slide was haematoxylin & eosin (H&E) stained and coverslipped for histological confirmation of tumor histological type, and presence or absence of tumor, by an expert lung pathologist (MNK). Five adjacent 10 μm slides were cut, H&E stained, and tumor or non-tumor material was manually microdissected. DNA was extracted via proteinase K digestion [58]. Briefly, cells were lysed in a solution containing 100 mM Tris-HCl (pH 8.0), 10 mM EDTA (pH 8.0), 1 mg/mL proteinase K, and 0.05 mg/mL tRNA and incubated at 50°C overnight. The DNA was bisulfite converted as previously described [59]. All studies were institutionally approved by the University of Southern California Institutional Review Board (IRB# HS-016041, HS-06-00447), and the identities of patients were not made available to laboratory investigators.

### Methylation analysis

DNA methylation analysis was done by MethyLight as previously described [59]. A pre-screen methylation analysis using cell lines and five sets of paired SQ/non-tumor adjacent lung (distinct from the samples used in this study) were used to screen over 300 DNA methylation loci, and led to the identification of 42 loci of interest, which were evaluated in this study. The primer and probe sequences are described in the supplemental data [see additional file 1]. In addition to primer and probe sets designed specifically for the locus of interest, two internal reference primer and probe sets directed against collagen and *ALU *repeats were included in the analysis to normalize for input DNA [60,61]. The percentage methylated reference (PMR) compares the level of methylation in the sample to *in vitro *methylated control DNA. It is calculated by dividing the GENE:reference ratio of a sample by the GENE:reference ratio of M. SssI-treated *in vitro *methylated human DNA and multiplying by 100 [59]. PMRs were individually calculated using the collagen and *ALU *controls and then averaged.

### Statistical analysis

Using PMR as a continuous variable, methylation levels of tumor samples were compared to adjacent non-tumor lung by means of the Wilcoxon signed rank test. The large number of loci analyzed increases the potential for false discovery. To counteract this risk, a multiple comparisons threshold was set and applied to those loci for which no previous data demonstrated their methylation in SQ of the lung at the time of analysis (Table 1, last column; [29]). To examine whether tumor-specific hypermethylation was seen in early as well as later stages of SQ lung cancer, methylation levels in tumor and adjacent non-tumor tissue were compared for "early" (stages IA and IB, n = 31) and more advanced cancers (stages II and III, n = 12), as well as for each individual stage (IA, IB, IIB and IIIA) using the Wilcoxon test. The same test was applied to the comparison of methylation levels in *tumor *samples between the early and advanced cancers. Associations with gender and age were tested using the Wilcoxon test to compare methylation levels within the tumor sample collection only. As an indicator of the potential utility of methylation of these loci as a marker for cancer, Receiver Operating Characteristic (ROC) curves were calculated for each of our top markers, using the PMR values for the tumor and adjacent non-tumor lung specimens. All statistical tests were two-sided. Statistical tests were carried out using JMP (v 5.0.1a, SAS Institute Inc, NC).

To determine which combinations of markers would be most effective to correctly identify tumor vs. non-tumor samples, we fit a random forest classifier to the data set, using the R programming language (v 2.5; [62]) and 90 samples and 42 variables. Using bootstrap samples of the data, we grew a forest of 30,000 trees. Splits were determined using a random sample of five variables and trees were grown until there was only one observation in each leaf. We determined error rates using the observations that were not used to generate the trees. For each observation, its outcome was predicted by having the majority vote from the trees that were generated without the original data point in their bootstrap sample. These predicted values were compared against the true tissue type to estimate prediction error.

## Competing interests

IALO and PWL are shareholders of Epigenomics AG, which has a commercial interest in the development of DNA markers for disease detection and diagnosis. None of the work performed in the laboratories of the authors is or has been supported or directed by Epigenomics.

## Authors' contributions

PPA was involved in experimental execution and extensive data analysis, drafting the manuscript, and generation of figures. JSG was involved in marker design, experimental execution and initial analysis. MNK reviewed all histological slides prior to microdissection. JAH provided samples and statistical discussions. ST helped locate and section tissues from the Los Angeles County Hospital and provided the linked and de-identified clinicopathological information. MC and DJW provided experimental advice and designed several of the MethyLight reactions used in this study. PWL provided experimental advice and discussion regarding data interpretation. KDS oversaw statistical analysis and drafted statistical sections of the manuscript. IALO designed the study, oversaw all aspects of the project, mentored PPA and JSG, and revised manuscript drafts. All authors reviewed and commented on the manuscript during its drafting and approved the final version.

## Supplementary Material

Additional file 1 Primer and Probe information and sequences. Click here for file
