# Supplementary material for: Identification of a panel of sensitive and specific DNA methylation markers for squamous cell lung cancer
Source: Mol Cancer. 2008 Jul 10;7:62. doi: 10.1186/1476-4598-7-62 (PMC2483990; doi:10.1186/1476-4598-7-62)
Supplement: Additional file 1 — Primer and Probe information and sequences. [file 1476-4598-7-62-S1.doc]

Additional File 1: Primer/Probe information and sequences

| **Gene Name** | **Reaction ID** | **Forward primer sequence** | **Reverse primer sequence** | **Probe sequence (5' with 6FAM and 3' with BHQ1)** |
| --- | --- | --- | --- | --- |
| ABCB1 | HB-051 | TCGGGTCGGGAGTAGTTATTTG | CGACTATACTCAACCCACGCC | 6FAM-ACGCTATTCCTACCCAACCAATCAACCTCA-BHQ-1 |
| CACNA1G | HB-162 | GTCGTCGGCGTTATTTTAGAAAGTT | CACCGACGCCCAACACA | 6FAM-ACGCTCCGCTCCCGAATACCCA-BHQ-1 |
| CCND2 | HB-040 | GGAGGGTCGGCGAGGAT | TCCTTTCCCCGAAAACATAAAA | 6FAM-CACGCTCGATCCTTCGCCCG-BHQ-1 |
| CDX1 | HB-195 | TGAGCGGTTGTTCGTCGTC | AAATCCCCCGCGCATACTA | 6FAM-CCTAAAACCGCCGCTACCGACCG-BHQ-1 |
| CPVL | HB 427 | ATTTACGTAGGGTAGGCGGTATTTAC | AACGCTACAAAAACAACCGACTAA | 6FAM-ATCCTTACGCGCCGCGACTCAA-BHQ-1 |
| CYP1B1 | HB 238 | GGGTTAGCGGTTGTTGAGGTAAC | CCGCGTTTCCGATCAATAA | 6FAM-AAAACGCGAACCGAAACTACCGCCT-BHQ-1 |
| DIRAS3 | HB-043 | GCGTAAGCGGAATTTATGTTTGT | CCGCGATTTTATATTCCGACTT | 6FAM-CGCACAAAAACGAAATACGAAAACGCAAA-BHQ-1 |
| DLEC | HB 225 | TCGTTGCGTATTTAAGATATTTCGTATT | CGTAACGCTCATTCTCGCTACC | 6FAM-TAATCAAACTTACGCTCACTTCGTCGCCG-BHQ-1 |
| GATM | HB 401 | TTTTTGTAGTCGCGTTTCGTTTC | GCCGACCCCACCACCTAT | 6FAM-CGACAACCAATAAAACCGCGAAAAACGA-BHQ-1 |
| GDNF | HB 222 | TCGTTTGTTCGCGTAGGTGTC | CGATATAAAACAACACCAAACAAACAAC | 6FAM-TCCCATAACTTCATCTTAAAATCCCGTCCG-BHQ-1 |
| GP1BB | HB-398 | TGGGAGCGGAAGTTTGAGC | AACGCGCGCTACAACGAC | 6FAM-AATAACACACTATCGCCGAAAACCGCAAA-BHQ-1 |
| GRIN2B | HB 250 | GTCGGATTTACGCGTCGAGT | CTACCGCCGCGCTAAAATAC | 6FAM-ACGCACGAAACTTCACCTACAACGTATCG-BHQ-1 |
| HIC1 | HB-168 | GTTAGGCGGTTAGGGCGTC | CCGAACGCCTCCATCGTAT | 6FAM-CAACATCGTCTACCCAACACACTCTCCTACG-BHQ-1 |
| HOXC9 | HB 440 | GGGAGTTGCGCGATCG | CTTCTCCTCTTTATACTTACTACCGACA | 6FAM-CGCGTCCGCCTCGAACGAAAAC-BHQ-1 |
| RASSF1 | HB-144 | GAGCGATGACGGAATATAAGTTGG | CGTCCACAAAATAATTCTAAATCAACTA | 6FAM-CACTCTTACCCACACCGCCGACG-BHQ-1 |
| LTB4R | HB-070 | GCGTTGGTTTTATCGGAAGG | AAACCGTAATTCCCGCTCG | 6FAM-GACTCCGCCCAACTTCGCCAAAA-BHQ-1 |
| MGMT | HB-159 | CTAACGTATAACGAAAATCGTAACAACC | AGTATGAAGGGTAGGAAGAATTCGG | 6FAM-CCTTACCTCTAAATACCAACCCCAAACCCG-BHQ-1 |
| MINT1 | HB-161 | GGGTTGAGGTTTTTTGTTAGCG | CCCCTCTAAACTTCACAACCTCG | 6FAM-CTACTTCGCCTAACCTAACGCACAACAAACG-BHQ-1 |
| MT1G | HB-204 | CGTTTAAGGGATTTTGTATTTGGTTTAT | CCGCTAAATCCGCACCG | 6FAM-CGCGATCCCGACCTAAACTATACGCA-BHQ-1 |
| MTHFR | HB-058 | TGGTAGTGAGAGTTTTAAAGATAGTTCGA | CGCCTCATCTTCTCCCGA | 6FAM-TCTCATACCGCTCAAAATCCAAACCCG-BHQ-1 |
| NEUROD1 | HB 259 | GTTTTTTGCGTGGGCGAAT | CCGCGCTTAACATCACTAACTAAA | 6FAM-CGCGCGACCACGACACGAAA-BHQ-1 |
| NEUROG1 | HB 261 | CGTGTAGCGTTCGGGTATTTGTA | CGATAATTACGAACACACTCCGAAT | 6FAM-CGATAACGACCTCCCGCGAACATAAA-BHQ-1 |
| ONECUT2 | HB 243 | CGTTACGTATATCGCGCGG | CAAAAACCTCCTXXXATAAACGACGAAT | 6FAM-AACACGCAATTACGCGCTTTTATACGCA-BHQ-1 |
| OPCML | HB-209 | CGTTTCGAGGCGGTATCG | CGAACCGCCGAAATTATCAT | 6FAM-AACAACTCCATCCCTAACCGCCACTTTCT-BHQ-1 |
| PAX-8 | HB-210 | ATCGATCGGTTTTATTTCGTTGAG | ACCAATCCGCGACCTACG | 6FAM-ACCTCGCCAAACCCATCTCCCAAAA-BHQ-1 |
| PENK | HB-163 | GGTTAATTATAAAGTGGTTTTAGTAGTCGG | CAACGTCTCTACGAAATCACGAAC | 6FAM-AACGCCTACCTCGCCGTCCCG-BHQ-1 |
| PITX2 | HB 234 | GGAGTGACGTGACGTTAGTAGAGATTT | AACCGCGCAACCGAACT | 6FAM-CGCCCGCGCGCCACTATACA-BHQ-1 |
| PLAGL1 | HB-199 | ATCGACGGGTTGAATGATAAATG | CTCGACGCAACCATCCTCTT | 6FAM-ACTACCGCGAACGACAAAACCCACG-BHQ-1 |
| PTPRN2 | HB 392 | MGTTUUAAUAGUTTMGGGTUUAGTUAUAT | AACTRCKCTTTCTCARCKCCTC | 6FAM-TAAAACGACCGCGTACTCGCCAAAAAA-BHQ-1 |
| **Gene Name** | **Reaction ID** | **Forward primer sequence** | **Reverse primer sequence** | **Probe sequence (5' with 6FAM and 3' with BHQ1)** |
| RARRES1 | HB 322 | GGCGAGTCGGATCGGAA | CGCAAACTCCTACAACAAACGA | 6FAM-CGCGCGACGCTTCACTTCTTCAA-BHQ-1 |
| RNR1 | HB-071 | CGTTTTGGAGATACGGGTCG | AAACAACGCCGAACCGAA | 6FAM-ACCGCCCGTACCACACGCAAA-BHQ-1 |
| RPA 3 | HB 104 | AGCGCGATTGCGATTTAGG | TTTCTCGACACCAATCAACGAA | 6FAM-TCCAACTTCGCCAATTAAATACGCGAAA-BHQ-1 |
| SEZ6L | HB-184 | GCGTTAGTAGGGAGAGAAAACGTTC | ATACCAACCGCCTCCTCTAACC | 6FAM-CCGTCGACCCTACAAAATTTAACGCCA-BHQ-1 |
| SFRP1 | HB-201 | GAATTCGTTCGCGAGGGA | AAACGAACCGCACTCGTTACC | 6FAM-CCGTCACCGACGCGAAAACCAAT-BHQ-1 |
| SFRP2 | HB 279 | TTTATAATTTTGATTTTTTTACGGTATTGG | GAAACCGCCTCGACGAACT | 6FAM-CTCGAATCTCCAACCACCGTTCAACAA-BHQ-1 |
| SLC38A4 | HB 430 | GATTTGAGGACGCGGGC | TTCCCCCGCGAAAACTAACT | 6FAM-CGCGACCGCCCGAAATCCTACT-BHQ-1 |
| TCF21 | HB 359 | GAGAGTTTTAATTGCGAGAATGGG | TCTTCTTAATAAACGCCTTCCTCC | 6FAM-CCAAACCGCCGCGACCCTTCT-BHQ-1 |
| TFAP2A | HB 314 | CGTTAATTTTTAAAGTATTTTTATGGATCG | CCGACAACCAACACTTTACGC | 6FAM-CGAAACCGAAAAAAACATATCCGTTCACG-BHQ-1 |
| TMEFF2 | HB-274 | CGACGAGGAGGTGTAAGGATG | CAACGCCTAACGAACGAACC | 6FAM-TATAACTTCCGCGACCGCCTCCTCCT-BHQ-1 |
| TNFRSF25 | HB-080 | GCGGAATTACGACGGGTAGA | ACTCCATAACCCTCCGACGA | 6FAM-CGCCCAAAAACTTCCCGACTCCGTA-BHQ-1 |
| TWIST | HB-047 | GTAGCGCGGCGAACGT | AAACGCAACGAATCATAACCAAC | 6FAM-CCAACGCACCCAATCGCTAAACGA-BHQ-1 |
| WDR33 | HB 435 | GTTATTACGTATTGGCGGGACG | ACGCAAATCGAACCTCACAAA | 6FAM-CCGCGATCCAAACGCGCG-BHQ-1 |
